# Colorectal cancer-promoting activity of the senescent peritoneal mesothelium

**DOI:** 10.18632/oncotarget.4932

**Published:** 2015-08-06

**Authors:** Justyna Mikuła-Pietrasik, Patrycja Sosińska, Konstantin Maksin, Małgorzata Kucińska, Hanna Piotrowska, Marek Murias, Aldona Woźniak, Dariusz Szpurek, Krzysztof Książek

**Affiliations:** ^1^ Department of Pathophysiology, Poznań University of Medical Sciences, Poznań, Poland; ^2^ Department of Clinical Pathology, Poznań University of Medical Sciences, Poznań, Poland; ^3^ Department of Toxicology, Poznań University of Medical Sciences, Poznań, Poland; ^4^ Division of Gynecological Surgery, Poznań University of Medical Sciences, Poznań, Poland

**Keywords:** cellular senescence, gastrointestinal cancers, mesothelial cells, mice xenografts, peritoneal cavity

## Abstract

Gastrointestinal cancers metastasize into the peritoneal cavity in a process controlled by peritoneal mesothelial cells (HPMCs). In this paper we examined if senescent HPMCs can intensify the progression of colorectal (SW480) and pancreatic (PSN-1) cancers *in vitro* and *in vivo*. Experiments showed that senescent HPMCs stimulate proliferation, migration and invasion of SW480 cells, and migration of PSN-1 cells. When SW480 cells were injected i.p. with senescent HPMCs, the dynamics of tumor formation and vascularization were increased. When xenografts were generated using PSN-1 cells, senescent HPMCs failed to favor their growth. SW480 cells subjected to senescent HPMCs displayed up-regulated expression of transcripts for various pro-cancerogenic agents as well as increased secretion of their products. Moreover, they underwent an epithelial-mesenchymal transition in the Smad 2/3-Snail1-related pathway. The search for mediators of senescent HPMC activity showed that increased SW480 cell proliferation was stimulated by IL-6, migration by CXCL8 and CCL2, invasion by IL-6, MMP-3 and uPA, and epithelial-mesenchymal transition by TGF-β1. Secretion of these agents by senescent HPMCs was increased in an NF-κB- and p38 MAPK-dependent mechanism. Collectively, our findings indicate that in the peritoneum senescent HPMCs may create a metastatic niche in which critical aspects of cancer progression become intensified.

## INTRODUCTION

The peritoneal cavity is a common site for metastasis of colorectal and pancreatic carcinomas [1]. The intraperitoneal spread of a malignancy has a poor prognosis and is generally treated as a manifestation of terminal disease [2]. With regard to colorectal tumors, the peritoneum is a second, to the liver, distal location to be colonized by cancer cells [3]. It has been estimated that 40–80% of patients who died from the disease developed intraperitoneal tumors [4]. The same case holds for advanced stages of pancreatic cancer, when 70–80% of nonresectable patients suffered from peritoneal metastases [5]. Peritoneal dissemination of gastrointestinal cancers typically proceeds in two ways: as a result of direct cell detachment from a primary tumor (along with bowel wall penetration in the case of colon carcinoma), or iatrogenically, due to incomplete resection of the primary tumor and/or malignant cell leakage from dissected blood and lymph channels [1, 5, 6].

There is general agreement that effective progression of a malignancy within the peritoneal cavity is related to the interplay between invading cancer and peritoneal mesothelial cells (HPMCs) [7]. The most recognized aspect of these interactions is cancer cell adhesion to the peritoneum, which has been found to be controlled by the binding of cancer cell surface ligand CD43 to the HPMC-derived intercellular adhesion molecule-1 (ICAM-1) [8, 9]. The strength of cell attachment has been recognized as being determined by local inflammation, in particular the activity of IL-1β and TNFα [10, 11], and by oxidative stress [12, 13]. Moreover, HPMCs are known to secrete a plethora of angiogenic agents that create a metastatic niche, thus facilitating tumor development [14, 15]. Interestingly, both the pro-adhesive and pro-angiogenic capabilities of HPMCs have been found to be markedly pronounced when the cells approach the end of their replicative lifespan and become senescent [12, 16, 17].

The role of cellular senescence in the progression of primary and metastatic tumors has become a matter of debate since the time it was discovered that senescent f ibroblasts stimulate the growth of pre-malignant and malignant breast cancer cells *in vitro* and the development of solid tumors in laboratory animals *in vivo* [18]. Until today, the pro-tumorigenic activity of senescent fibroblasts has also been revealed towards melanoma [19], prostate [20], lung [21], and cervical cancer cells [22]. A detailed analysis of the senescent cell phenotype allowed to establish that their capacity to stimulate cancer cell expansion is underlined by increased secretion of numerous agents (cytokines, chemokines, growth factors, extracellular matrix-remodeling molecules) that create a specific, inflammation-like environment in which vital elements of cancer cell behavior are intensified [23].

In the study presented here we comprehensively examined whether senescent HPMCs, which are known to accumulate in the peritoneal cavity [24], may promote the progression of colorectal and pancreatic carcinomas *in vitro* and stimulate the development of peritoneal tumors in a mice xenograft model *in vivo*. Phenomenological assessments, covering measurements of cancer cell proliferation, migration, invasion and epithelial-mesenchymal transition as well as analysis of xenograft growth dynamics and its vascularization, were followed by mechanistic studies in which soluble mediators of senescent HPMC activity and the signaling pathways underlying their production were delineated. In addition, molecular events occurring in cancer cells at both mRNA and protein levels in response to the activity of senescent HPMCs were analyzed here.

## RESULTS

### Senescent HPMCs promote proliferation, migration and invasion of SW480 cells, and migration of PSN-1 cells *in vitro*

**Figure 1:**
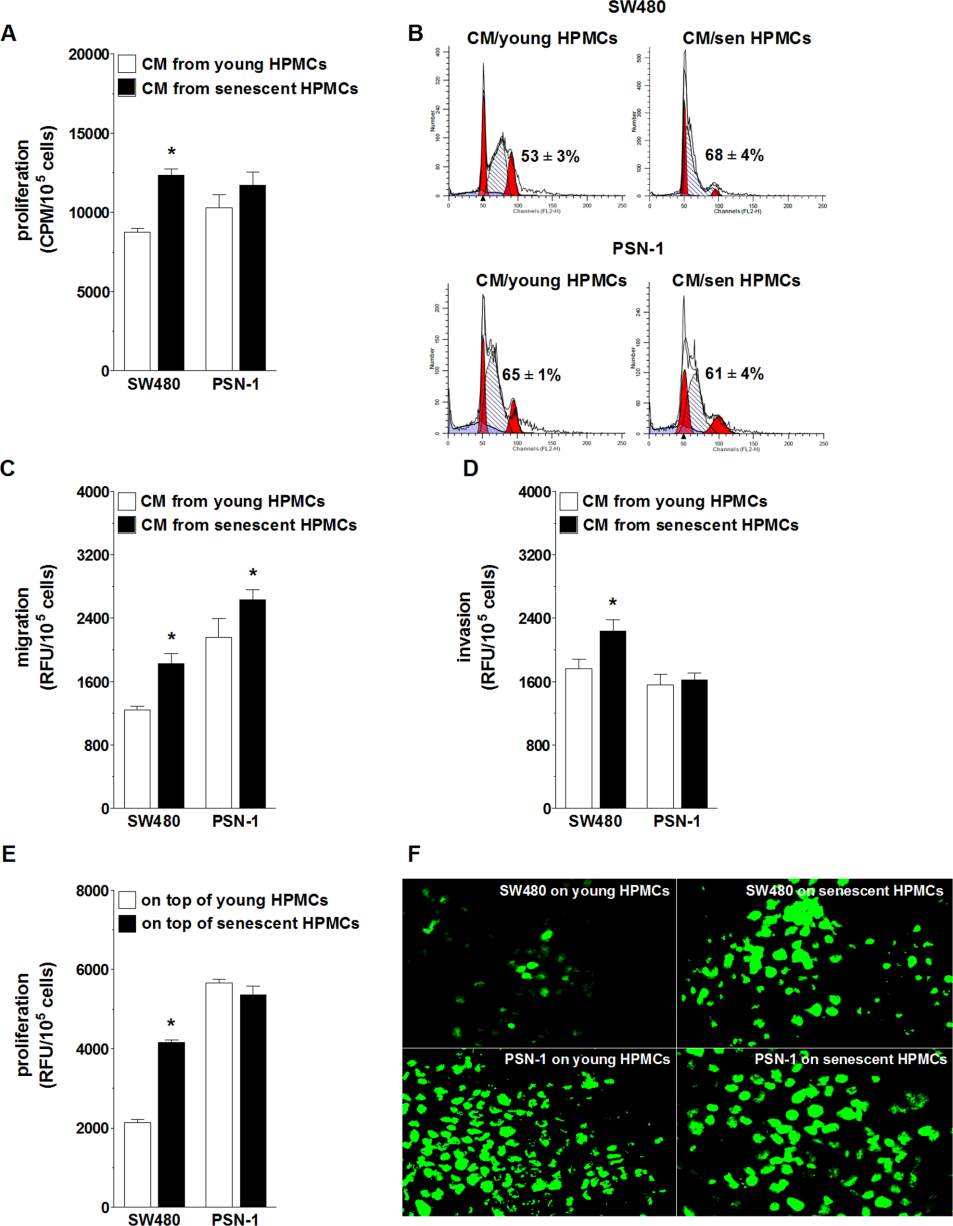
Effect of senescent HPMCs on the progression of SW480 and PSN-1 cancer cells *in vitro* Cancer cell proliferation **A.** migration **C.** and invasion **D.** were examined upon their exposure to conditioned medium (CM) generated by young and senescent (sen) HPMCs. Panel **B.** shows representative histograms of cancer cell distribution in particular phases of the cell cycle (corresponding to the panel A). Phase S, in which cells actively replicate their DNA, is shown as the hatched area. The numbers above this area express the percentage of cells in the S-phase. Cancer cell proliferation was also analyzed upon the cells’ direct physical contact with young and senescent HPMCs **E.** Panel **F.** shows representative results of a fluorescence-based proliferation assay in which SW480 and PSN-1 cells proliferated on top of the feeder layer formed by HPMCs treated with mitomycin C (corresponding to the panel E; magnification x400). The asterisks indicate significant differences (*P* < 0.05 for A, C, D; *P* < 0.03 for E) as compared with cells exposed to CM from young HPMCs or grown on top of young HPMCs. The experiments were performed using primary cultures of HPMCs obtained from 8 different donors. RFU: Relative Fluorescence Units; CPM: Counts Per Minute. The cancer cells were used in hexaplicates. The results are expressed as mean ± SD.

The effect of senescent HPMCs on the progression of colorectal (SW480) and pancreatic cancer (PSN-1) cells was evaluated with regard to a cancer cell response to soluble agents released to the environment (conditioned medium; CM) by the HPMCs, and to their reaction to direct physical contact with HPMCs in a co-culture. Experiments focusing on the role of soluble agents showed that SW480 cells subjected to CM from senescent HPMCs proliferated, migrated and invaded the Matrigel much more efficiently than their counterparts exposed to CM from young HPMCs. In contrast, PSN-1 cells undergoing treatment with senescent HPMC-derived CM displayed improved migratory properties as compared with cells incubated with CM from young HPMCs, whereas their capacity to proliferate and invade the Matrigel remained unchanged (Fig. 1A, 1C, 1D). An improvement in SW480 cell proliferative potential upon their exposure to CM from the senescent HPMCs coincided with an increased percentage of cells in the S phase of the cell cycle. In the case of PSN-1 cells, the fraction of the cells in the S phase was unchanged in both experimental groups (Fig. 1B).

When it comes to the role of cell-cell interactions, SW480 cells seeded on top of a feeder layer established from senescent HPMCs divided more vigorously than cells growing on young HPMCs. Under the same conditions, the proliferation rate of PSN-1 cells seeded on young and senescent HPMCs appeared to be comparable (Fig. 1E, 1F).

### Senescent HPMCs induce an epithelial-mesenchymal transition in SW480 cells

**Figure 2:**
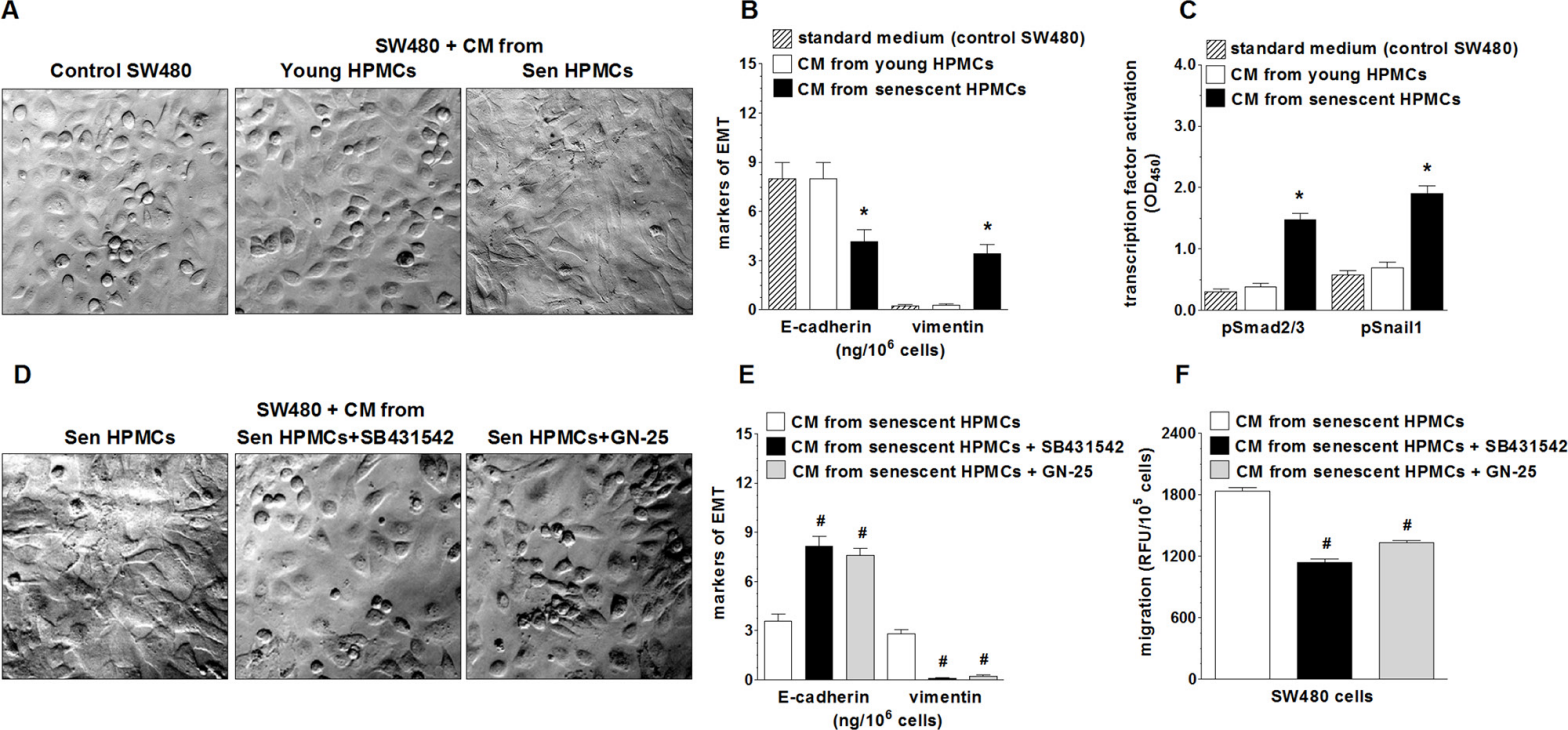
Effect of senescent HPMCs on the development of EMT in SW480 cells The cancer cells were exposed to standard growth medium (control SW480) and to conditioned medium (CM) from young or senescent (sen) HPMCs, and then their morphology (a shift into the spindle-shaped appearance; magnification x400) **A.** and the concentration of E-cadherin and vimentin in the cell lysates **B.** were evaluated. Panel **C.** shows the effect of senescent HPMCs on the activation (by phosphorylation) of transcription factors Smad2/3 and Snail1. Panel **D.** shows representative pictures of the loss of the EMT phenotype by cancer cells which were pre-incubated with inhibitors of Smad 2/3 (SB431542) and Snail1 (GN-25). The effect of Smad2/3 and Snail1 inhibition on the concentration of E-cadherin and vimentin in SW480 cells **E.** and on the migration of SW480 cells **F.** The asterisks indicate a significant difference (*P* < 0.04 for B and *P* < 0.01 for C) as compared with cells exposed to CM from young HPMCs, while the hashes show a significant difference (*P* < 0.02 for E and *P* < 0.03 for F) as compared with cells subjected to CM from senescent HPMCs (without cancer cell pre-incubation with transcription factor inhibitors). The experiments were performed using primary cultures of HPMCs obtained from 8 different donors. The cancer cells were used in hexaplicates. The results are expressed as mean ± SD.

In order to examine whether increased motility of SW480 cells incubated in the presence of CM from senescent HPMCs was related to the development of the epithelial-mesenchymal transition (EMT), cancer cell morphology and the expression of E-cadherin, a marker of epithelial cells, and vimentin, a marker of mesenchymal cells [25], in cell lysates were analysed. The study showed that SW480 cells exposed to CM generated by senescent HPMCs became spindle-shaped, in contrast to their counterparts subjected to CM from young HPMCs or the standard growth medium, which maintained a characteristic, epithelial-like appearance (Fig. 2A). At the same time, the level of E-cadherin in these cells was remarkably decreased while the level of vimentin was elevated (Fig. 2B). Similar experiments performed with PSN-1 cells showed that the morphology of the cancer cells exposed to CM from senescent HPMCs remained squamous, and that the level of E-cadherin and vimentin in these cells was unaltered (not shown).

Because the development of EMT often involves Smad 2/3 and Snail1 [26], activation of these transcription factors upon cancer cell treatment with a senescent HPMC-derived medium was examined. The experiments showed that the level of phosphorylated Smad 2/3 and Snail1 in cancer cells subjected to senescent HPMCs was significantly increased (Fig. 2C). At the same time, when the cancer cells were pre-incubated with specific Smad 2/3 (SB431542) and Snail1 (GN-25) inhibitors, their further exposure to CM derived from senescent HPMCs failed to reverse their morphology into being spindle-shaped (Fig. 2D), to decrease the concentration of E-cadherin, to increase the concentration of vimentin (Fig. 2E), and to improve their migratory capabilities (Fig. 2F).

### Senescent HPMCs stimulate the dynamics at which intraperitoneal colorectal tumors develop *in vivo*

**Figure 3:**
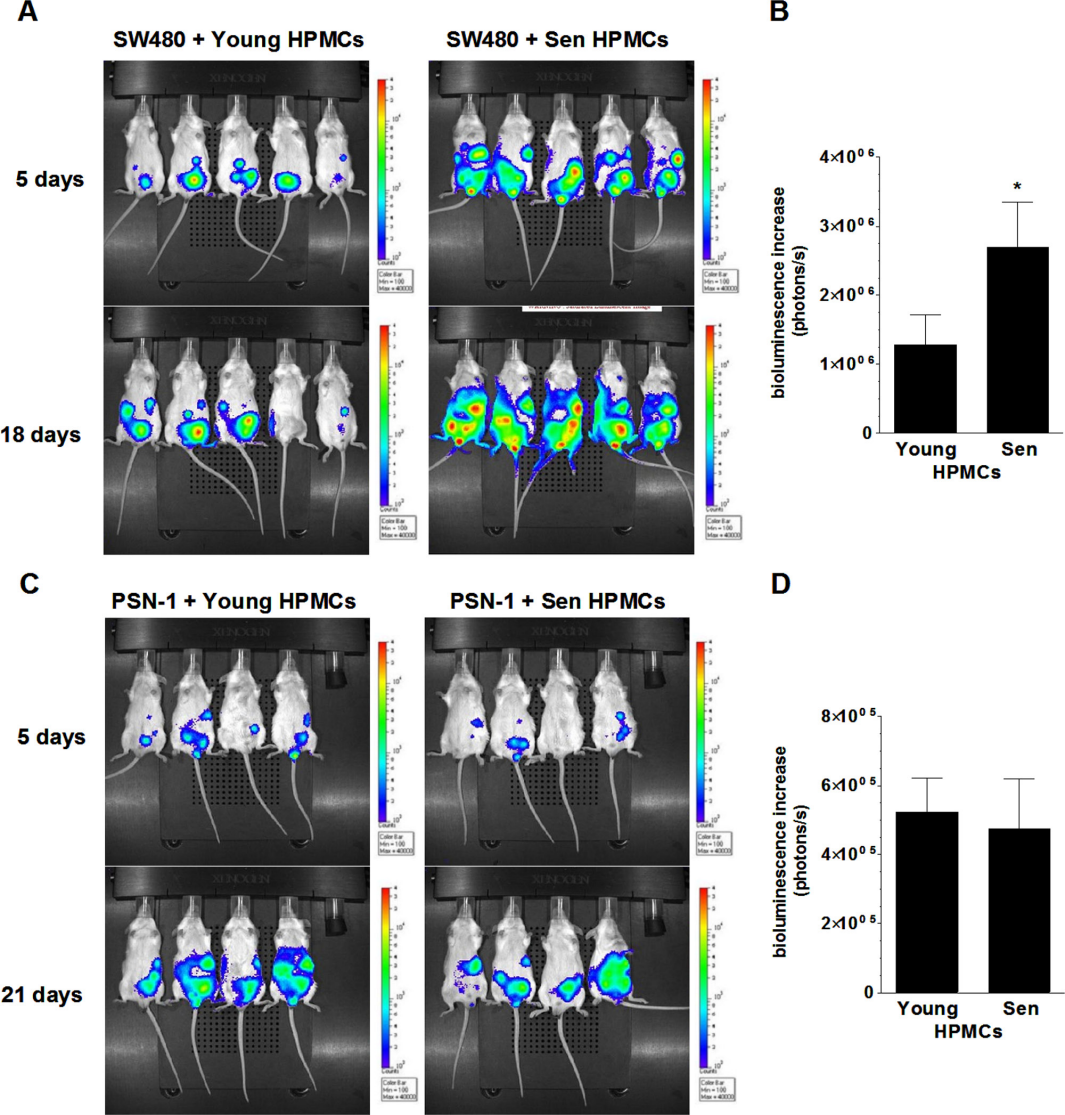
Bioluminescence-based examination of the intraperitoneal development of colorectal (A, B) and pancreatic (C, D) tumors in mice injected with SW480 and PSN-1 cancer cells, respectively, in combination with young or senescent (sen) HPMCs Representative images showing bioluminescence intensity recorded 5 and 18 (SW480) or 21 (PSN-1) days after cell implantation **A, C.** The dynamics of xenograft development, estimated according to the difference between the highest bioluminescence intensity recorded throughout the experiment and the initial value, were recorded 5 days after cell injection **B, D.** The asterisks indicate a significant difference (*P* < 0.05) as compared with xenografts established in the presence of young HPMCs. Experiments were performed on 6 animals per group with HPMCs established from 6 different donors. The results are expressed as mean ± SD.

In order to examine the effect of senescent HPMCs on the development of peritoneal colorectal and pancreatic tumors *in vivo*, immunocompromised Scid mice were injected i.p. with mixtures of luciferase-transfected SW480 and PSN-1 cells with young or senescent HPMCs (1:1). Afterwards, the development of xenografts was monitored according to cancer cell bioluminescence recorded using the IVIS Spectrum within 18 (SW480) and 21 days of the experiment (PSN-1). The study showed that the dynamics at which the xenografts developed upon SW480 cancer cell injection together with the senescent HPMCs was considerably higher than in the case when the cancer cells were accompanied by young HPMCs (Fig. 3A, 3B). At the same time, in experiments conducted with PSN-1 cells, senescent HPMCs failed to improve tumor growth dynamics as compared with young cells (Fig. 3C, 3D).

**Figure 4:**
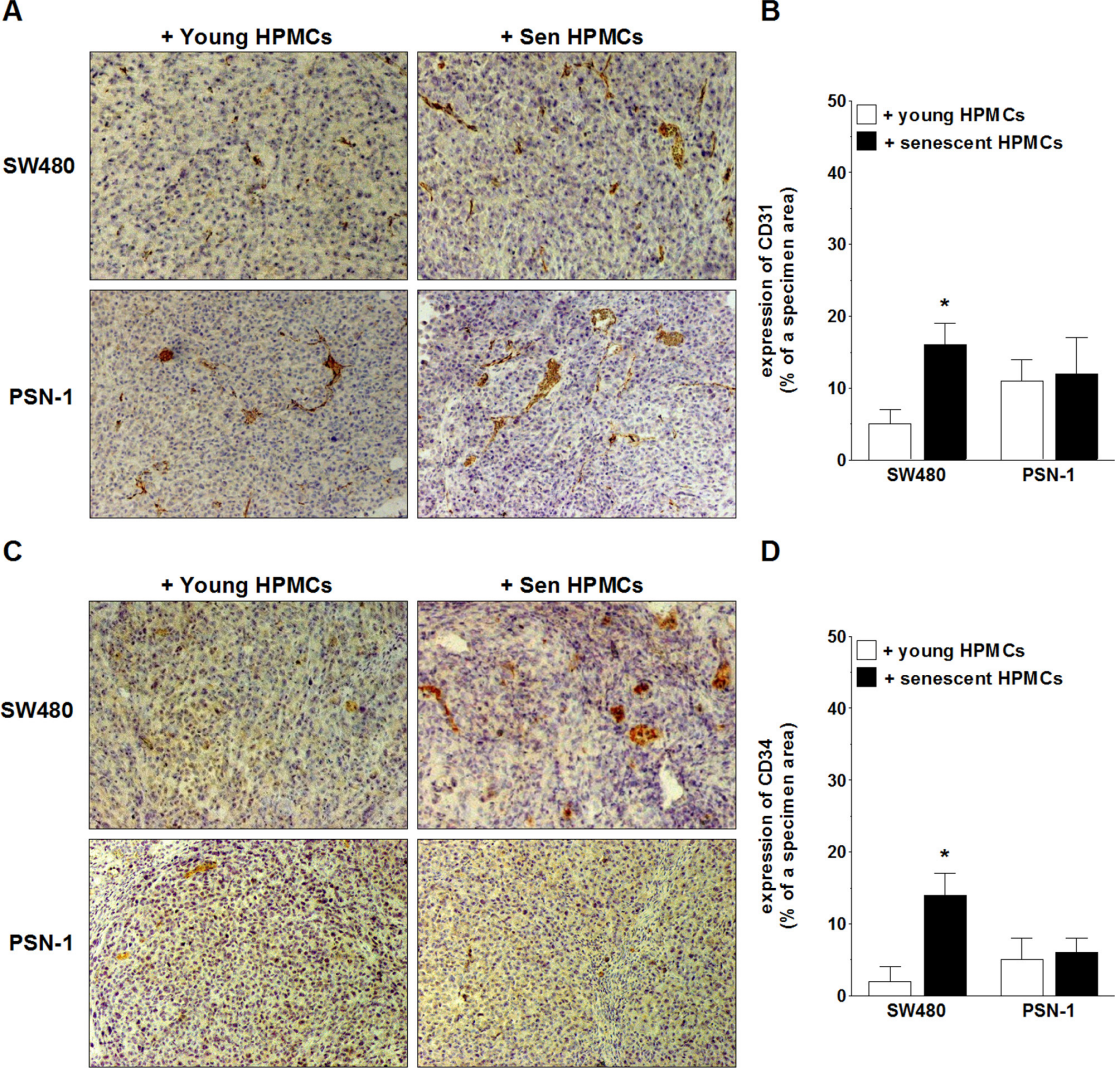
Microscopic evaluation of tumor vascularization of xenografts excised from the mouse peritoneal cavity after i.p. injection of SW480 and PSN-1 cells together with young or senescent (sen) HPMCs The development of microvessels was analyzed according to immunohistochemical reactions against CD31 **A, B.** and CD34 **C, D.** i.e. markers of endothelial cells. Panels **A** and **C.** show the representative results of CD31 and CD34 staining (the brown color indicates a positive reaction; magnification x100). Panels **B** and **D.** show the results of the quantification of the brown-stained area reflecting the presence of CD31- **B.** and CD34-positive cells **D.** The results are expressed as a percentage (%), and the whole area of a specimen is considered to be 100%. The asterisks indicate a significant difference (*P* < 0.04) as compared with the xenografts that developed in the presence of young HPMCs. The experiments were performed on 6 animals per group with HPMCs established from 6 different donors. The results are expressed as mean ± SD.

In order to examine the magnitude of xenograft vascularization, the excised tumors were subjected to standard H+E staining (in order to confirm their cancerous nature; not shown) and then to an immunohistochemical reaction towards markers of vascular endothelial cells, CD31 and CD34 [27, 28]. The study showed that the expression of both antigens in xenografts formed by SW480 cells in the presence of senescent HPMCs was considerably greater as compared with tumors that had developed upon cancer cell co-injection with young cells. The analysis of tumors generated by PSN-1 cells administered together with senescent HPMCs did not exhibit a difference in the expression of CD31 or CD34 as compared with their counterparts established in the presence of the young cells (Fig. 4).

Because in the case of SW480 cells the senescent HPMCs stimulated all of the studied aspects of their progression, and in the case of PSN-1 cells there were no obvious effects of their activity, all subsequent experiments were performed on colorectal cancer cells only.

### SW480 cells subjected to senescent HPMCs display up-regulated expression of genes and increased secretion of proteins that contribute to cancer progression

**Figure 5:**
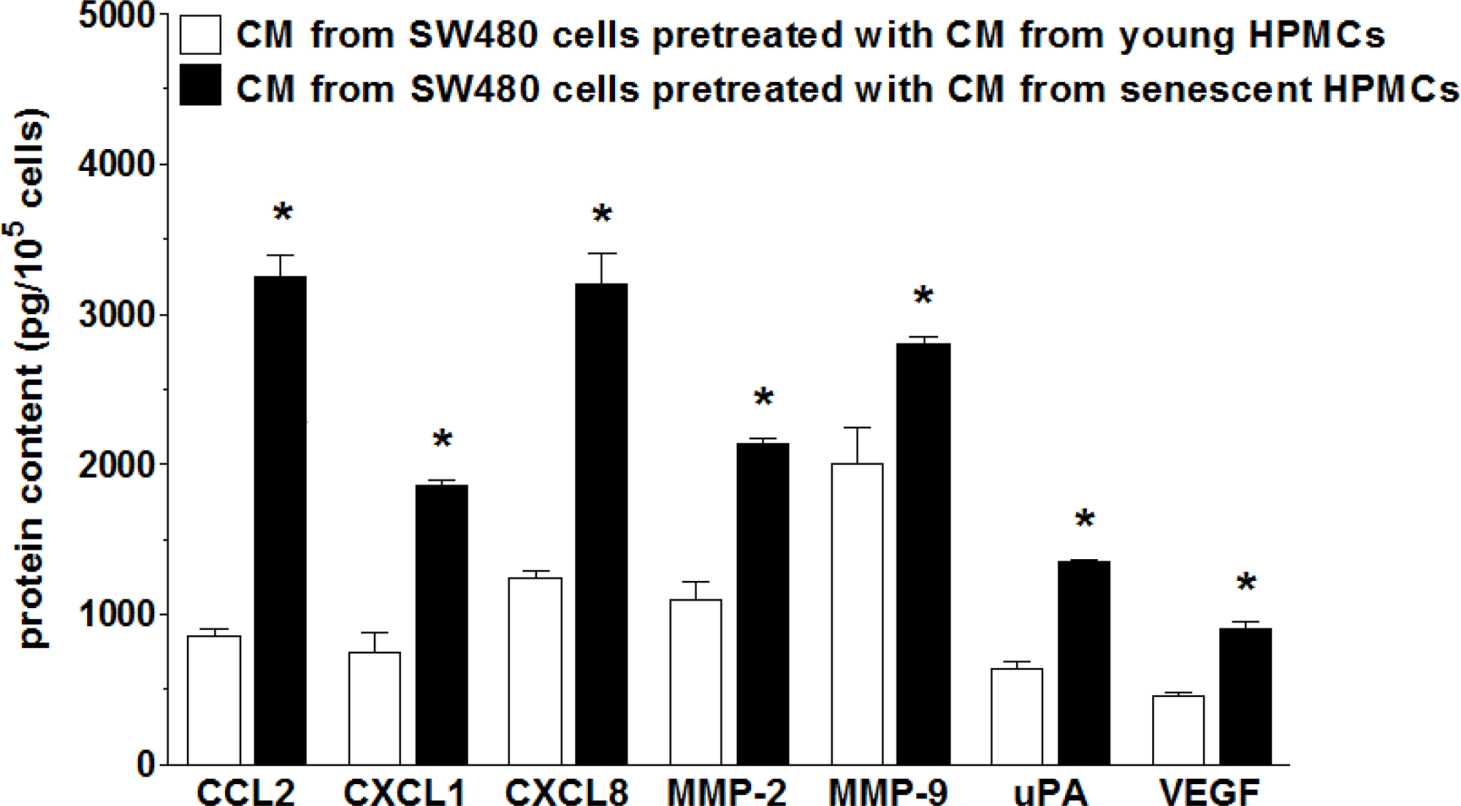
Secretory profile of SW480 cells subjected to conditioned medium (CM) generated by young and senescent HPMCs The asterisks indicate a significant difference (*P* < 0.01 for CCL2, CXCL1, CXCL8; *P* < 0.03 for MMP-2 and uPA; *P* < 0.05 for MMP-9 and VEGF) as compared to cells exposed to CM from young HPMCs. The experiments were performed using primary cultures of HPMCs obtained from 12 different donors. The cancer cells were used in hexaplicates. The results are expressed as mean ± SD.

Upon establishing that senescent HPMCs promote the proliferation/migration/invasion of SW480 cancer cells *in vitro* and the development of intraperitoneal tumors *in vivo*, the cancer cells were again subjected to CM from senescent HPMCs in order to analyze changes in the expression of transcripts (quantitative PCR) and secretion (ELISA) of their products, which are known to be involved in cancer progression. As is shown in Table 1, colorectal cancer cells undergoing the activity of senescent HPMCs display up-regulated mRNA for several genes involved in cell cycle progression, angiogenesis, inflammation and extracellular matrix (ECM) remodeling. Moreover, an analysis of autologous conditioned medium generated by SW480 cells pre-treated with CM from senescent cells revealed that they secrete increased levels of such important mediators of carcinogenesis as CCL2, CXCL1, CXCL8, MMP-2, MMP-9, uPA and VEGF to the environment (Fig. 5).

**Table 1:** Genes whose expression in SW480 cancer cells increased upon their exposure to conditioned medium (CM) generated by senescent HPMCs

Function	Gene	Fold change
**cell cycle progression and proliferation**	CCL20	5.2 ± 0.4
	cell division cycle 2	2.3 ± 0.1
	cyclin A2	3.4 ± 0.2
	cyclin B1	3.5 ± 0.6
	cyclin E	5.2 ± 0.6
	Ki67	3.1 ± 0.3
	PCNA	4.1 ± 0.6
**angiogenesis**	CXCL1	2.1 ± 0.4
	CXCL8	3.2 ± 0.6
	FGF2	4.6 ± 0.2
	HGF	4.1 ± 0.5
	HIF-3α	5.2 ± 0.3
	VEGF	2.5 ± 0.2
**inflammation**	CCL2	5.2 ± 0.4
	IL-1R	3.2 ± 0.3
	IL-6R	2.1 ± 0.3
**ECM remodeling**	ADAM	2.1 ± 0.1
	MMP-2	5.7 ± 0.3
	MMP-9	3.3 ± 0.6
	TIMP-1	3.4 ± 0.2
	PAI-1	3.2 ± 0.4
	u-PA	6.4 ± 0.5

SW480 cells exposed to CM from senescent HPMCs were compared with their counterparts subjected to CM from young HPMCs and treated as the control group. The calculation of relative change in mRNA was standardized to the GAPDH housekeeping gene. The results come from experiments conducted with mesothelial cells obtained from 6 different donors and are expressed as means ± SD.

### Senescent HPMCs produce agents that may stimulate the progression of colorectal cancer

In order to examine if primary HPMCs used in the current experiments are characterized by the so-called senescence-associated secretory phenotype (SASP), RNA isolated from young and senescent HPMCs as well as the conditioned medium (proteins) generated by these cells were subjected to investigations towards agents that may contribute to improved cancer cell expansion. An analysis of gene expression in the HPMCs showed that senescent cells display up-regulated expression of several transcripts coding for proteins involved in angiogenesis, inflammation and ECM synthesis/remodeling (Table 2). The greatest increase was observed with respect to the angiogenesis mediators CXCL1 and VEGF, the pro-inflammatory agents CCL2 and IL-6, and ECM constituent, fibronectin, and ECM remodeling factors PAI-1 and TGF-β. Expression of some of the examined transcripts, including FGF2, CXCL5, CTGF, MMP-12 and vitronectin, remained unchanged.

**Table 2:** Comparative quantitative PCR analysis of young and senescent HPMCs with regard to expression of genes that may be involved in cancer progression

Function	Gene	Young cells	Senescent cells	*P*-value
**angiogenesis**	angiopoietin-1	0.5 ± 0.2	1.3 ± 0.1	*P* < 0.05
	CXCL1	1.2 ± 0.3	3.1 ± 0.2	*P* < 0.04
	CXCL8	0.3 ± 0.04	1.4 ± 0.3	*P* < 0.02
	FGF2	1.1 ± 0.3	1.1 ± 0.2	NS
	VEGF	0.8 ± 0.3	4.1 ± 0.5	*P* < 0.02
**inflammation**	CCL2	0.3 ± 0.04	2.4 ± 0.04	*P* < 0.03
	CXCL5	0.4 ± 0.06	0.3 ± 0.04	NS
	IL-6	0.9 ± 0.05	3.5 ± 0.4	*P* < 0.01
	ICAM-1	0.5 ± 0.04	2.1 ± 0.03	*P* < 0.02
**synthesis and remodeling of ECM**	collagen I	0.5 ± 0.05	1.6 ± 0.2	*P* < 0.03
	collagen IX	0.8 ± 0.02	0.9 ± 0.02	NS
	CTGF	0.5 ± 0.2	0.3 ± 0.05	NS
	fibronectin	0.9 ± 0.05	4.4 ± 0,3	*P* < 0.03
	MMP-3	0.5 ± 0.04	1.8 ± 0.07	*P* < 0.03
	MMP-12	0.9 ± 0.06	0.9 ± 0.04	NS
	PAI-1	0.4 ± 0.06	2.9 ± 0.05	*P* < 0.02
	TGF-α	0.3 ± 0.03	0.3 ± 0.03	NS
	TGF-β1	0.4 ± 0.06	3.6 ± 0.2	*P* < 0.01
	TIMP-1	0.3 ± 0.3	0.4 ± 0.3	NS
	TSP-1	0.4 ± 0.02	2.5 ± 0.5	*P* < 0.03
	u-PA	0.5 ± 0.01	1.7 ± 0.1	*P* < 0.04
	vitronectin	0.6 ± 0.2	0.6 0.2	NS

The calculation of relative change in mRNA was standardized to the GAPDH housekeeping gene. The results come from experiments conducted with mesothelial cells obtained from 12 different donors and are expressed as means ± SD.

**Figure 6:**
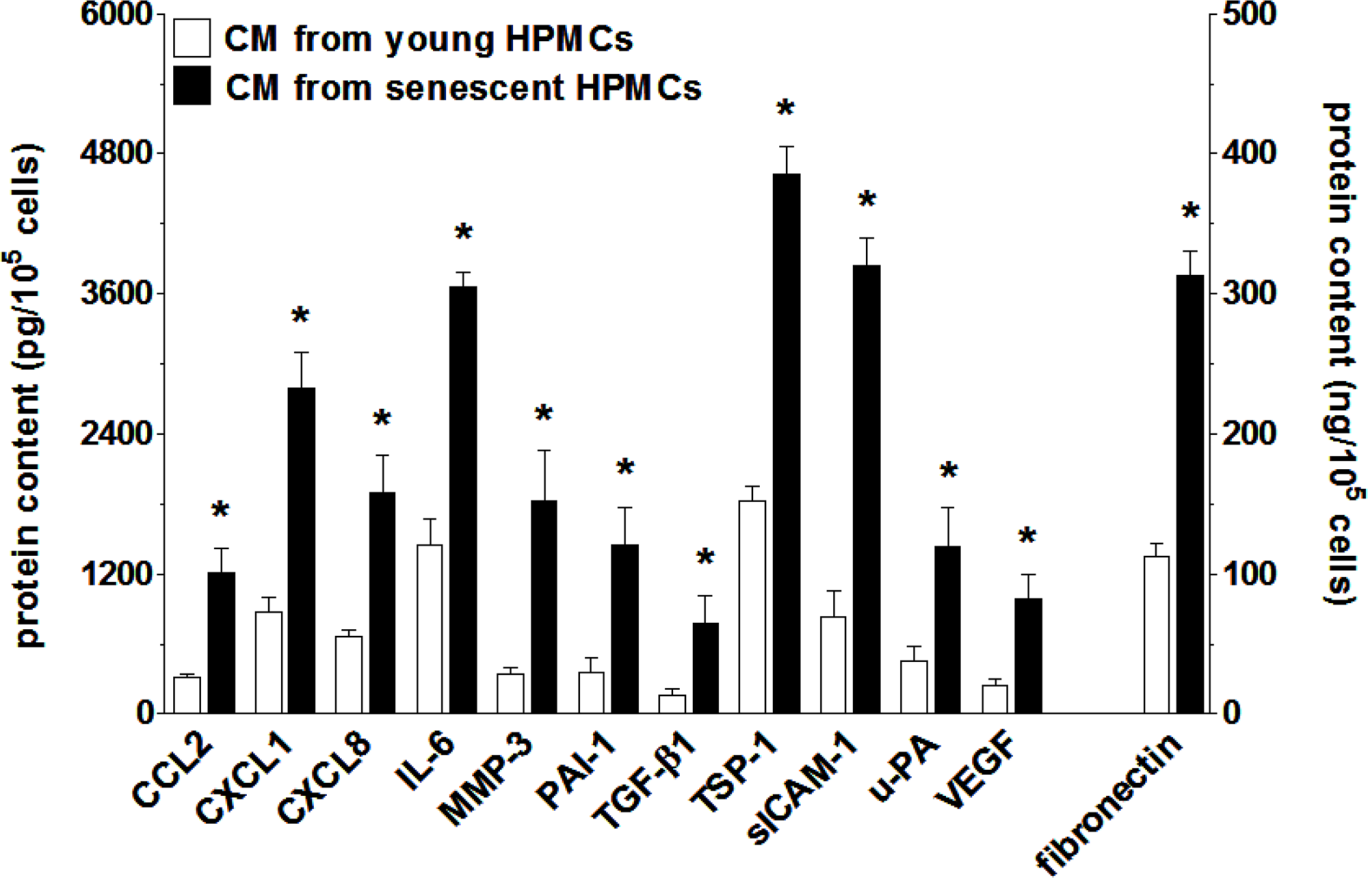
Increased secretion of various proteins involved in cancer cell progression by senescent HPMCs The concentration of soluble forms of these agents was examined in samples of conditioned medium (CM) generated by young and senescent HPMCs. The asterisks indicate significant differences (*P* < 0.01 for CCL2, CXCL1, CXCL8, IL-6, MMP-3, PAI-1, TGF-β1, sICAM-1, VEGF; *P* < 0.03 for TSP-1, uPA, fibronectin) as compared with CM generated by young HPMCs. The experiments were performed using primary cultures of HPMCs obtained from 12 different donors. The results are expressed as mean ± SD.

Analysis of SASP at the mRNA level was followed by analysis of the senescent HPMCs’ secretome. To this end, products of the previously analyzed genes that are released in a soluble form to a culture environment were quantified in conditioned medium from young and senescent HPMCs. The study showed that the senescent cells were characterized by increased secretion of 12 out of 17 measured proteins (Fig. 6). In fact, the results obtained were consistent with the qPCR analysis, as the levels of angiopoietin-1, CXCL5, FGF2, TGF-α and TIMP-1 appeared to be unchanged (not shown).

### Secretory phenotype of senescent HPMCs is controlled by the complementary activation of NF-κB and p38 MAPK

**Figure 7:**
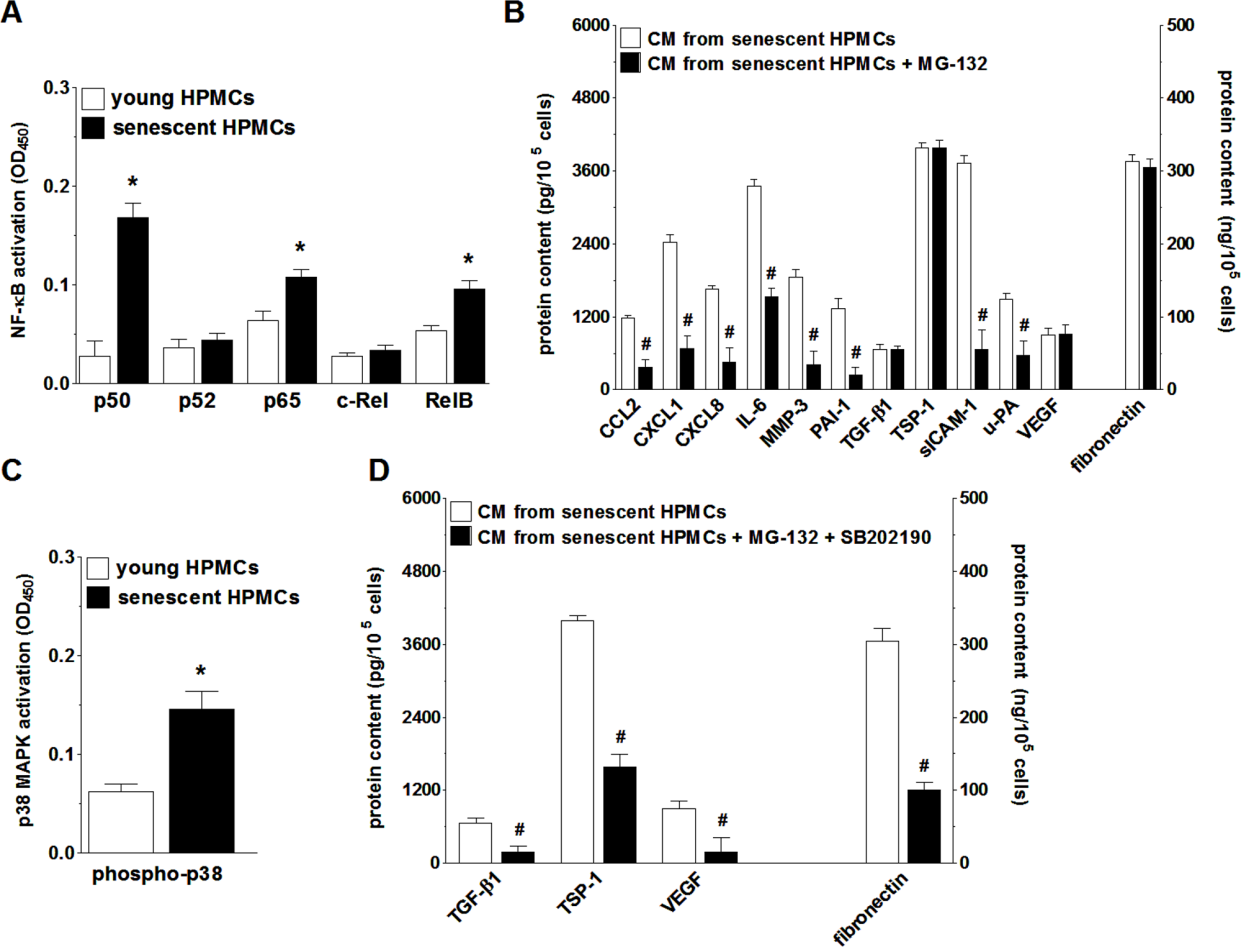
Analysis of signaling pathways underlying increased secretory properties of senescent HPMCs Activation of transcription factor NF-κB subunits in young and senescent HPMCs **A.** Changes in the senescent HPMCs’ secretory properties upon their pre-incubation of the NF-κB inhibitor, MG-132 **B.** Changes in p38 MAPK activation (by phosphorylation) during senescence of HPMCs **C.** Effect of simultaneous inhibition of NF-κB and p38 MAPK (using SB202190) on the secretory capabilities of senescent HPMCs **D.** The asterisks indicate significant differences (*P* < 0.03 for A and *P* < 0.04 for C) as compared with young HPMCs. The hashes indicate significant differences (*P* < 0.02 for B and *P* < 0.04 for D) as compared with CM obtained from senescent HPMCs (without the blockade of NF-κB or p38 MAPK). The experiments were performed in duplicates using primary cultures of HPMCs obtained from 8 different donors. The results are expressed as mean ± SD.

In order to verify if NF-κB, known to be a master regulator of the transcription of SASP genes [29], controls this trait also in HPMCs, the activation of five NF-κB subunits (p50, p52, p65, c-Rel, and RelB) during senescence of HPMCs was analyzed. Afterwards the secretome of these cells upon NF-κB inhibition was evaluated. The experiments showed that senescent HPMCs displayed increased expression of three out of the five NF-κB subunits studied, i.e. p50, p65 and RelB (Fig. 7A). Interestingly, when the transcriptional activity of NF-κB was blocked by the sequential pre-incubation of middle-aged HPMCs with MG-132, the intensified production of 8 out of the 12 SASP components (CCL2, CXCL1, CXCL8, IL-6, MMP-3, PAI-1, uPA, sICAM-1) by senescent HPMCs was efficiently prevented (Fig. 7B).

Because the inhibition of NF-κB did not suppress SASP entirely, further studies were conducted to identify an additional signaling element involved in this feature development. To this end, senescence-related activation and effects of inhibition were investigated with regard to p38 MAPK, a molecule known to be one of the most important NF-κB up-stream regulators and cellular senescence effectors [29]. These studies showed that the phosphorylation level of p38 MAPK in replicatively senescent HPMCs was significantly increased (Fig. 7C). At the same time, combined inhibition of NF-κB and p38 MAPK in the middle-aged cells resulted in reduced secretion of those proteins whose level in the CM from the senescent HPMCs remained unchanged upon the exclusive inhibition of NF-κB (Fig. 7D).

### Hypersecretion of IL-6, CXCL8, CCL2, MMP-3, uPA and TGF-β1 by senescent HPMCs underlines increased aggressiveness of SW480 cells

**Figure 8:**
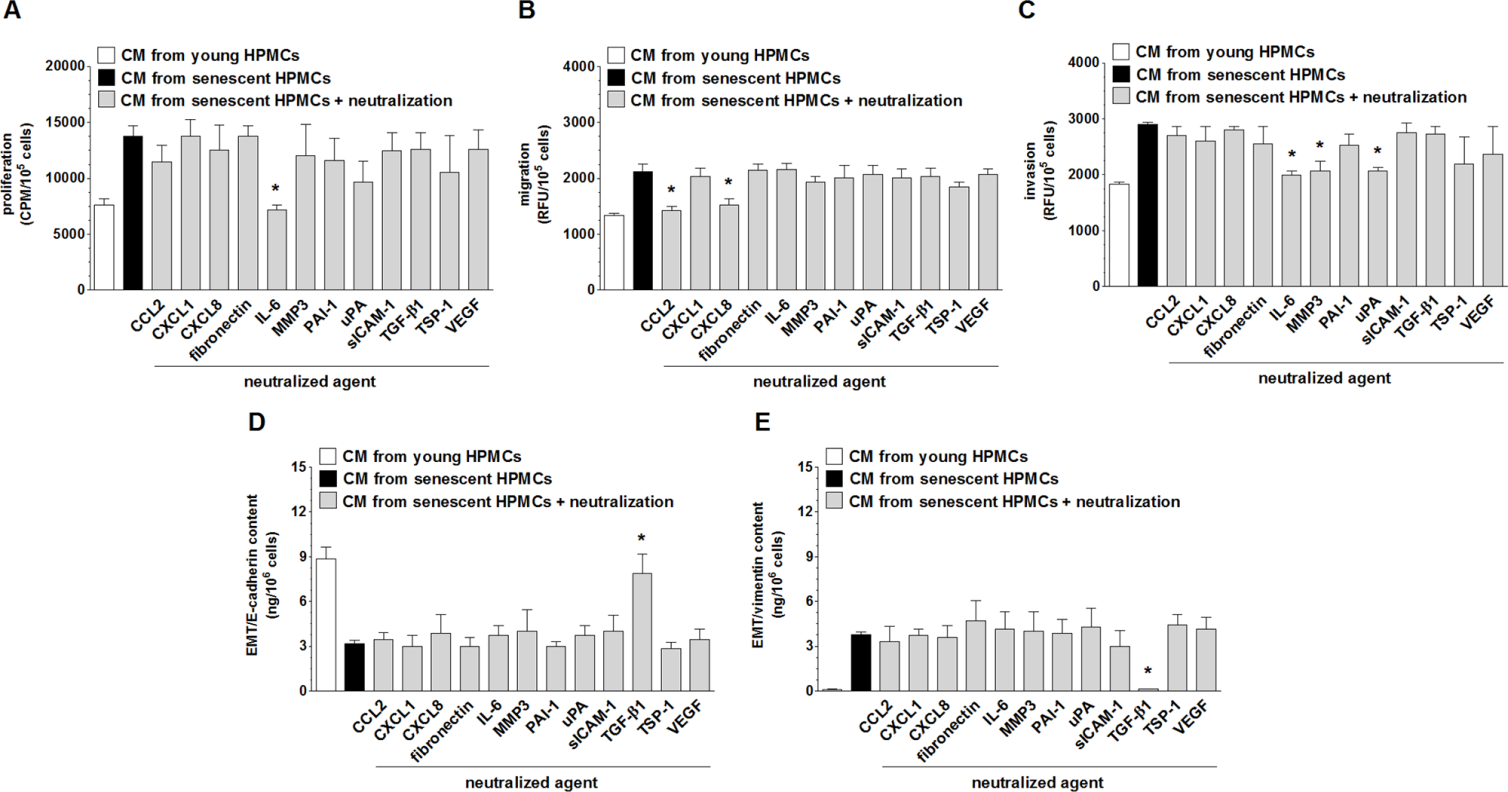
Identification of the mediators of pro-cancerogenic activity of senescent HPMCs Proliferation **A.** migration **B.** invasion **C.** and the concentration of E-cadherin **D.** and vimentin **E.** in SW480 cancer cells were analyzed upon the neutralization of proteins secreted at increased concentrations to the environment by senescent HPMCs. The asterisks indicate significant differences (*P* < 0.02 for A and E; *P* < 0.05 for B and C; *P* < 0.04 for D) as compared with CM from senescent HPMCs. The experiments were performed using primary cultures of HPMCs obtained from 8 different donors. The cancer cells were used in hexaplicates. RFU: Relative Fluorescence Units; CPM: Counts Per Minute. The results are expressed as mean ± SD.

Because senescent HPMCs were found to stimulate four aspects of SW480 cell progression *in vitro*, i.e. their proliferation, migration, invasion and EMT, intervention studies with specific neutralizing antibodies or other agents that specifically neutralize the activity of particular mediators were performed to identify which elements of the senescent HPMCs’ secretome were responsible for the pro-cancerous activity of these cells. The experiments showed that intensification of each element of aggressive cancer cell behavior upon the activity of senescent HPMCs was mediated by diverse proteins. Augmented proliferation of the cancer cells was reduced to values characterizing the effect of young HPMCs by the neutralization of IL-6. Increased cancer cell migration was suppressed by the inhibition of CCL2 and CXCL8. Increased cancer cell invasion was reduced upon the inhibition of IL-6, MMP-3 and u-PA. Last but not least, development of the EMT (evaluated according to the concentration of E-cadherin, initially down-regulated by senescent HPMCs and vimentin, initially up-regulated by senescent cells) was uniformly prevented by the neutralization of TGF-β1 (Fig. 8). Significantly, in the case of migration and invasion, which were supported by two and three different agents, respectively, simultaneous neutralization of all these mediators led to a situation in which cancer cells exposed to CM from senescent HPMCs migrated and invaded the Matrigel at a similar efficiency as their counterparts exposed to CM from young cells (data not shown).

## DISCUSSION

Over the past decade, much has been discovered about the possible contribution of senescent cells to the progression of primary and metastatic tumors [18, 19, 21, 22]. At the same time, an overwhelming majority of existing data derive from studies on the pro-cancerogenic activity of senescent fibroblasts; very sparse reports concern similar activity of other somatic cell types [30]. The range of malignancies examined in this respect is also very limited.

In this paper, we are the first to report that senescent human peritoneal mesothelial cells (HPMCs), which constitute a dominant cell population within the peritoneal cavity [31] and are known to accumulate with age [24], actively promote three critical aspects of colorectal cancer cell (SW480) aggressiveness, i.e. proliferation, migration and invasion. These findings extend our previous observations that senescent HPMCs stimulate adhesion of SW480 cells via intensified ICAM-1-CD43 interactions [17]. Our current study revealed that the cancer growth-promoting activity of senescent HPMCs may occur either through soluble agents released by these cells to the environment (conditioned medium) or by direct, physical contact between HPMCs and the cancer cells. The effect exerted by the soluble mediators appeared to be more pronounced, which distinguishes HPMCs from fibroblasts, whose growth-promoting activity towards breast cancer cells proceeds primarily through direct cell-cell interactions [18]. This profound role of the senescent HPMCs’ secretome seems to be a characteristic feature of these cells, as it also drives (in contrast, again, to fibroblasts [32]) senescence spreading among their proliferating neighbors [33].

Having found increased motility, in particular invasiveness, of cancer cells subjected to senescent HPMCs, further studies were aimed at establishing if this effect may be related, at least to some extent, to the epithelial-mesenchymal transition (EMT); a process in which cancer cells lose their uniform epithelial-like appearance and become more plastic and aggressive [34]. The experiments showed that, indeed, SW480 cells subjected to CM from senescent HPMCs adopted a spindle-shaped morphology and displayed, simultaneously, a declined level of E-cadherin—a marker of epithelial cells and increased level of vimentin—a marker of mesenchymal cells [25]. The ability of senescent HPMCs to elicit EMT in malignant cells stays in agreement with earlier studies on fibroblasts, in which senescent cells, conversely to non-senescent cells, secreted factors capable of inducing EMT in two non-aggressive breast cancer cell lines. Also in that model, senescence-dependent EMT was accompanied by decreased expression of E-cadherin and increased expression of vimentin [35].

When it comes to a plausible mechanism of EMT development in SW480 cells, the zinc-finger transcription factor Snail1 and its transcriptional up-regulators Smad2/3, acting together as the master mediators of EMT, were evaluated [26]. The tests revealed that senescent HPMCs activated Smad2/3-Snail1 signaling in SW480 cells, which eventually conferred EMT appearance. This conclusion stems from observations that the inhibition of either Smad2/3 or Snail1 in the cancer cells prevents EMT occurrence and that cells subjected to this manipulation displayed a restored level of E-cadherin, known to be prone to be repressed by Snail1 [36], and a declined concentration of vimentin. Under the same regimen, the migratory capabilities of SW480 cells were significantly diminished.

The reliability of the pro-cancerogenic effects of senescent HPMCs *in vitro* confirmed the experiments done *in vivo* showing that the dynamics of colorectal tumor development in a mice peritoneal cavity upon SW480 cell co-injection together with senescent HPMCs was markedly improved. The intensified intraperitoneal progression of colorectal tumors in the presence of senescent HPMCs was plausibly related not only to increased adhesion, proliferation, migration, invasion or EMT of the cancer cells, but also to increased tumor vascularization. The critical role of adequate angiogenesis in tumor progression was shown in previous studies on mice, in which various kinds of peritoneal tumors grew preferentially at sites rich in blood vessels [37]. Our observations are supported by these findings because the xenografts that developed at higher dynamics due to their fueling by senescent HPMCs displayed increased density of microvessels formed by CD31/CD34-positive endothelial cells [27, 28].

This increased angiogenesis within the cancerous tissue in response to senescent HPMCs may have two complementary reasons. The first reason is related to the ability of senescent cells to secrete increased amounts of various angiogenic stimuli, as was previously revealed in the case of fibroblasts [38]. Here, we also found that senescent HPMCs hypersecrete diverse angiogenesis mediators, including CCL2, CXCL1, CXCL8 and VEGF, which in combination with our previous experience concerning their biological activity [16] may indicate that they are truly able to support the formation of new blood vessels. This effect resembles, to some extent, the “reactive stroma” phenomenon in which cancer-associated fibroblasts create a permissive microenvironment in which all critical needs of an expanding tumor, including excessive oxygen and nutrient supply, are fully satisfied [39]. Increased xenograft vascularization was also found in the case of breast cancer when the malignant cells were co-transplanted in mice in combination with senescent fibroblasts [38].

The second mechanism of observed increased tumor vascularization may be associated with the modulating activity of senescent HPMCs on the cancer cell secretory profile. Namely, SW480 cells subjected to senescent HPMCs displayed up-regulated expression/production of the mRNA/protein for agents that mediate various elements of tumor expansion, e.g. cell cycle progression, inflammation, extracellular matrix (ECM) remodeling and angiogenesis. This may imply a scenario in which SW480 cells, initially modified by senescent HPMCs, support their own growth by sending mitogenic signals towards the neighboring vascular endothelium [40]. This mechanism may also be the case for the exacerbation of other elements of cancer progression, such as proliferation, migration, invasion and EMT, which were reinforced by the senescent HPMCs.

Concluding this part of the study, it should be stressed that in contrast to the explicit pro-tumorigenic activity of senescent HPMCs towards colorectal cancer, the same senescent cells failed to stimulate all but one (migration) of the investigated aspects of pancreatic cancer cell (PSN-1) aggressiveness. This is intriguing, as our previous experiments showed that senescent HPMCs stimulate pancreatic cancer cell adherence more effectively than their young counterparts [17]. It seems, however, that the pro-cancerogenic activity of senescent cells towards pancreatic carcinoma was restricted only to two *in vitro* processes (adhesion and migration) but did not translate to the stimulation of all necessary processes that underlie intensified pancreatic tumor formation *in vivo*. This may also indicate that the pro-cancerogenic activity of senescent HPMCs may proceed in a tumor-specific manner.

There is broad consensus that the cancerogenic potential of senescent cells is primarily associated with a unique feature of these cells, i.e. their “senescence-associated secretory phenotype” (SASP). Generally speaking, SASP refers to increased release by senescent cells of a myriad of agents that alter the tissue microenvironment in such a way that it becomes permissive for the critical element of cancer progression [23]. In fact, senescent HPMCs share this feature with other kinds of somatic cells, such as fibroblasts, normal epithelial cells and keratinocytes [35, 41], as they secrete to the environment (likely also *in vivo*) increased amounts of twelve proteins that promote angiogenesis, inflammation and ECM remodeling. Some of these agents are likely responsible for the stimulatory activity of senescent HPMCs on the progression of SW480 cells.

Before, however, the plausible mediator(s) of increased cancer cell proliferation, migration, invasion and EMT were identified, the signaling underlying SASP development in senescent HPMCs was delineated. According to present knowledge, SASP is controlled by the transcriptional activity of NF-κB, which may be activated by diversified stimuli, including DNA damage, p38 MAPK, inflammasomes and ceramides [29]. When analyzing SASP determinants in senescent HPMCs, studies were concentrated on five NF-κB subunits and on p38 MAPK, which has been found to underlie both the development of the senescence phenotype in HPMCs and the capacity of these cells to support cancer cell adhesion [17, 42]. Of the five NF-κB subunits studied, the activity of three, i.e. p50, p65 and RelB, appeared to be elevated in senescent HPMCs. At the same time, when NF-κB activation was inhibited in the course of HPMC senescence, increased secretion of the majority of SASP elements (CCL2, CXCL1, CXCL8, IL-6, MMP-3, PAI-1, ICAM-1, uPA) was effectively prevented. The inhibition of the remaining four SASP components (TGF-β1, TSP-1, VEGF and fibronectin) was achieved when both NF-κB and p38 MAPK were neutralized. The latter finding, pointing to the interplay between NF-κB and p38 MAPK during SASP development, is in agreement with a previous report showing that deletion of the p65 NF-κB subunit remarkably reduced the secretion of pro-inflammatory cytokines induced by p38 MAPK in the fibroblasts [43].

When it comes to the identification of SASP-related mediators of increased colorectal cancer cell proliferation, migration, invasion and EMT, the efficiency of these processes was studied upon the neutralization of individual proteins secreted in excess by senescent HPMCs. The experiments revealed that the mediators of each aspect of cancer cell behavior are diversified: proliferation was driven by IL-6, migration by CXCL8 and CCL2, invasion by IL-6, MMP-3 and uPA, and EMT by TGF-β1. Collectively, the cancer-promoting activity of senescent HPMCs appeared to be related to increased secretion and activity of seven different proteins.

Finding the colorectal cancer cell growth-promoting activity of IL-6 agrees with the literature data [44], which additionally indicate that this cytokine exerts its effects via transsignaling, in particular involving the activation of STAT3 [45]. Further studies with antibodies directed against this cytokine confirmed the contribution of IL-6 in SW480 cell progression, especially their clonogenicity [46]. As per cancer cell migration, CXCL8 is known to generate a chemotactic gradient stimulating the motility of colorectal carcinoma in a mechanism involving the expression of αVβ6 integrins [47]. CCL2, in turn, has not been linked with increased migration of colorectal malignancy, however, such activity of this chemokine has been reported in the case of breast [48] and ovarian cancer cells [49]. Apart from the stimulation of colorectal cancer cell proliferation, IL-6 has also been recognized as a molecule contributing to cancer cell invasiveness [46]. The pro-migratory capabilities of MMP-3 [50] and uPA [51], as well as the master role of TGF-β1 in the development of EMT [52], have also been described.

Taken together, our study is the first to show that senescent HPMCs contribute to increased intraperitoneal aggressiveness of colorectal cancer, and that this activity is associated with increased secretion by these cells of various proteins that mediate all critical elements of cancer cell progression *in vitro* and *in vivo*. The results obtained here may constitute a significant step in gaining a deeper understanding of the role of senescent cells (here HPMCs) that progressively accumulate in tissues during aging in the development of various age-related disorders, including cancer. Last but not least, the identification of cancer-promoting features of senescent HPMCs may be used to establish strategies that can then be used to prevent and/or to treat certain intraperitoneal pathologies by targeted modification of the HPMCs’ phenotype. Taking into account the latest successful attempts to use modified mesothelial cells to repair the peritoneal membrane [53], such plans seem to be worthy of consideration.

## MATERIALS AND METHODS

### Chemicals

Unless otherwise stated, all chemicals were purchased from Sigma (St. Louis, MO). Cell culture plastics were obtained from Nunc (Roskilde, Denmark). SB202190 (p38 MAPK inhibitor) and MG-132 (NF-κB inhibitor) were purchased from Cell Signaling Technology (Danvers, MA), SB431542 (Smad inhibitor) from Abcam (Cambridge, UK) and GN-25 (Snail inhibitor) from Cayman Chemical (Ann Arbor, MI). Neutralizing antibodies against CCL2, CXCL1, CXCL8, ICAM-1, IL-6, PAI-1, TGF-β1 and VEGF were from R&D Systems (Abingdon, UK). The antibody against TSP-1 was from Abcam (Cambridge, UK; anti-TSP-1). UK-356618 and BC 11 hydrobromide, selective inhibitors of MMP-3 and uPA, respectively, were obtained from R&D Systems. The GRGDSP peptide that blocks fibronectin from binding to its receptors was purchased from Sigma.

### Culture and senescence of peritoneal mesothelial cells

Human peritoneal mesothelial cells (HPMCs) were isolated from pieces of omentum, as described elsewhere [54]. The tissues were obtained from 12 patients undergoing elective abdominal surgery. The reasons for the surgery included hernia (7) and bowel obstruction (5). Informed consent has been obtained from all patients. The cultures were established from individuals with no evidence of peritonitis and no overt diabetes, uremia and peritoneal malignancy. The age of the donors ranged from 30 to 45 years old. The cells were identified as pure mesothelial by their typical cobblestone appearance at confluence and uniform positive staining for HBME-1 antigen. The cells were propagated in medium M199 supplemented with L-glutamine (2 mM), penicillin (100 U/ml), streptomycin (100 μg/ml), hydrocortisone (0.4 μg/ml), and 10% (v/v) fetal bovine serum (FBS) (Gibco™, Invitrogen, Karlsruhe, Germany). The cultures were maintained at 37°C in a humidified atmosphere of 95% air and 5% CO_2_. The biological features of the cells used for experiments, including their morphology, proliferative potential, senescence phenotype and secretory capabilities was uniform. The age of a patient and a reason of the surgery did not elicit any differences in HPMC characteristics.

HPMCs were forced to senescence by serial passaging at 7-day intervals with a fixed seeding density of 3 × 10^4^ cells/cm^2^. Cells from passages 1–2 were treated as ‘young’, while those that failed to increase in number during 4 weeks and stained > 70% for SA-β-Gal were considered as ‘senescent’ [55]. In some experiments, HPMCs that reached about 50% of their lifespan (later called “middle-aged” cells) were treated with SB202190 (10 μM) and/or MG-132 (10 μM) for 2 hours daily until the end of their ability to replicate. Between exposures, the cells were maintained in standard medium.

The study was approved by the Bioethics Commission at the Poznań University of Medical Sciences.

### Culture of cancer cells

Colorectal cancer cell line SW480 was purchased from the American Type Culture Collection (Rockville, MD, USA) and maintained in DMEM with L-glutamine (2 mM) and 10% FBS. Pancreatic cancer cell line PSN-1 was purchased from the European Collection of Cell Cultures (Porton Down, UK) and propagated in RPMI-1640 with 10% FBS. In some experiments, the cancer cells were pre-incubated (for 4 hours at 37°C with mixing) with SB431542 (10 μM) and GN-25 (10 μM), i.e. specific inhibitors of Smad2/3 and Snail1, respectively.

### Collection of conditioned media

Conditioned media (CM) were collected from young and senescent HPMCs. Briefly, 3 × 10^5^ of the cells was seeded into 25 cm^5^ flasks, allowed to attach for 4 hours and incubated in a serum-free medium for 72 h. In order to collect CM from the cancer cells, the cells were grown until reaching 80–90% confluency and then were maintained in serum-free conditions for 72 h. Samples of CM were centrifuged, filtered through a 0.2 μm pore size filter, and stored at −80°C until required.

### Examination of a cell secretory profile

The secretory properties of HPMCs and cancer cells were analyzed at mRNA (quantitative real-time PCR) and protein levels (ELISA). For qPCR, RNA was isolated from the cells using mini columns (GenElute^™^ Mammalian Total RNA Miniprep Kit). One microgram of total RNA was reverse-transcribed to cDNA using the random hexamers method. Taqman quantitative real-time PCR reactions were performed in triplicate according to manufacturer's instructions (Applied Biosystems, Hong Kong) in an ABI Prism 7700 Sequence Detection System using appropriate primer sets (Assays-on-Demand, Applied Biosystems). The calculation of relative change in mRNA was standardized to the housekeeping gene GAPDH.

Concentrations of CCL2, CXCL1, CXCL8, sICAM-1, IL-6, PAI-1, TGF-β1, TSP-1, MMP-3, u-PA and VEGF in cell culture supernatants were determined with appropriate DuoSet^®^ Immunoassay Development kits (R&D Systems). The concentration of fibronectin was estimated using the Fibronectin Human ELISA Kit, purchased from Abcam. All assays were performed according to manufacturer's instructions.

### Analysis of epithelial-mesenchymal transition (EMT)

Incidence of EMT in cancer cells undergoing exposure to conditioned medium obtained from young and senescent HPMCs was determined according to the concentration of E-cadherin and vimentin in cell lysates. Concentration of E-cadherin was determined with appropriate DuoSet^®^ Immunoassay Development kit purchased from R&D Systems whereas the concentration of vimentin was examined using PathScan^®^ Total Vimentin Sandwich ELISA Kit, obtained from Cell Signaling Technology.

### Proliferation, migration and invasion assays

Cancer cell proliferation in response to CM from the HPMCs was assessed in serum-free conditions on low-density cultures using the radioisotope method, essentially as described in [55]. The results of these investigations were expressed as CPM (Counts Per Minute) per 10^5^ cells. In some experiments, cancer cells were probed with a fluorescent dye, i.e. carboxyfluorescein diacetate succinimidyl ester (CFDA-SE; Bio-Rad, Hercules, CA) [56], then carefully washed twice to remove any excess of the dye, and then seeded on top of a feeder layer created by sub-confluent young or senescent HPMCs, which had been pre-treated with mitomycin C (10 μg/ml for 2 h at 37°C). Under these conditions, proliferation of the cancer cells was estimated according to green fluorescence emitted by the product of CFDA-SE conversion, i.e. CFSE. The results were recorded using the Wallac Victor 2 spectrofluorometer (Perkin-Elmer, Massachusetts, USA) and with a fluorescent microscope Zeiss Axio Obserwer D1 (Carl-Zeiss, Jena, Germany). The results were expressed as RFU (Relative Fluorescence Units) per 10^5^ cells.

Cell migration was examined using Transwell inserts (Costar, Inc., NY, USA), as described in [57]. Analysis of cancer cell invasion was performed using the BD BioCoat™ Tumor Invasion Chamber (BD Biosciences, Bedford, MA), as per manufacturer's instructions. In the migration and invasion assays, CM generated by young/senescent HPMCs was used as a chemoattractant source. In all assays, cancer cell exposure to PMC-derived CM or the HPMCs themselves lasted 24 h.

In some experiments, cancer cell proliferation, migration, invasion and EMT were analyzed in response to CM from senescent HPMCs, which were pre-incubated (for 4 h at 37°C with mixing) with antibodies against PAI-1 (20 μg/ml), TGF-β1 (400 ng/ml), IL-6 (200 ng/ml), CXCL1 (10 μg/ml), CXCL8 (20 μg/ml), VEGF (5 μg/ml), ICAM-1 (25 μg/ml), and TSP-1 (5 μg/ml), or with UK-356618 (5 nM) and BC 11 hydrobromide (10 μM), the inhibitors of MMP-3 and u-PA, respectively. In the same set of experiments, cancer cells were pre-incubated (for 4 h at 37°C with mixing) with GRGDSP (10 μM)—a peptide that specifically binds to the fibronectin receptor. The results of cell migration and invasion were expressed as RFU per 10^5^ cells.

### Cell cycle analysis

Cancer cells subjected to a conditioned medium from young and senescent HPMCs were harvested with trypsin-EDTA solution and fixed in ice-cold 70% ethanol overnight at −20°C. After washing with PBS, the cells were re-suspended in 0.1 M sodium citrate, pH 7.8 for 1 min and incubated for 30 min in PBS containing 5 mg/mL of propidium iodide (Molecular Probes, Eugene, OR, USA) and 0.1 mg/mL of RNase A. In order to determine cell distribution in the cell cycle, one million cells were analyzed using a FACSCalibur™ flow cytometer with ModFit LT™ software (Verity Software House, Topsham, ME, USA).

### Animal studies

The experiments were performed on immunocompromised Scid mice (CB17/ I cr-Prkdc/ I crI coCrl, Charles River, Wilmington, MA) which were injected i.p. with cancer cells mixed with young or senescent HPMCs (both 2 × 10^6^ cells) in 100 μl of sterile PBS. Before implantation, the cancer cells were stably transfected with plasmid for luciferase using Lipofectamine LTX with Plus Reagent (Invitrogen, Carlsbad, CA) and selected with Geneticin (Gibco, Eggenstein, Germany). The luciferase reporter gene encoding plasmid pGL4.51 was purchased from Promega (Madison, WI). Non-invasive tumor monitoring with the IVIS Spectrum imaging system (Caliper Life Sciences, Hopkington, MA) was performed according to manufacturer's protocol. The animals were kept in the experiment for either 18 days (SW480) or 21 days (PSN-1), and then were humanely sacrificed.

Tumors that developed in the peritoneal cavity were excised with a small margin of normal tissue, fixed in 4% paraformaldehyde, dehydrated in alcohol series, embedded in paraffin and cut into 4 μm sections in a microtome. In order to identify cancerous tissue, standard H+E staining was performed.

All procedures on laboratory animals were performed in compliance with the EU Directive 2010/63/EU. The study was approved by the Local Ethical Commission for Experiments on Animals.

### Tumor vascularization measurements

Tumor vascularization was examined according to immunohistochemical detection of endothelial cell markers, i.e. CD31 and CD34 [27, 28]. In brief, the specimens were incubated with an antibody against CD31 (Leica Biosystems, Buffalo Grove, IL) and against CD34 (Santa Cruz Biotechnology, Santa Cruz, CA), both diluted 1:25. Mouse lung preparations were used as the positive and negative controls. The staining was conducted using the Autostainer Link 48 (Dako, Carpinteria, CA). Planimetric analysis of the brown-stained area reflecting the presence of CD31/CD34-positive cells was conducted using ImageJ 1.47v (Wayne Rasband, National Institute of Health, USA). Six to eight x100 fields covering almost the whole of each of the six sections per group were examined. The results were expressed as a percentage (%), and the whole area of a specimen was treated as 100%.

### Signaling pathway analysis

Activation of nuclear factor kappaB (NF-κB) subunits was examined using the TransAM^®^ NF-κB Family kit (Active Motif, Carlsbad, CA). Activation of Smad2/3 was quantified using PathScan^®^ Phospho-Smad2/Smad3 (Ser465/467) Sandwich ELISA kits (Cell Signaling Technology). Activation of Snail1 was examined with the Phospho-SNAI1 Colorimetric Cell-Based ELISA Kit (Aviva Systems Biology, San Diego, CA). Activation of p38 Mitogen Activated Protein Kinase (MAPK) was quantified using a Fast Activated Cell-based ELISA (FACE™) p38 Kit (Active Motif). All assays were performed as per manufacturer's instructions.

### Statistics

Statistical analysis was performed using GraphPad Prism™ 5.00 software (GraphPad Software, San Diego, USA). The means were compared with the Wilcoxon and Mann-Whitney tests. Differences with a *P*-value < 0.05 were considered to be statistically significant.
